# Association between sulfur microbial diet and the risk of colorectal cancer precursors in older adults

**DOI:** 10.3389/fnut.2023.1167372

**Published:** 2023-08-14

**Authors:** Yi Xiao, Hongmei He, Ling Xiang, Haitao Gu, Zhiquan Xu, Haoyun Luo, Xiaorui Ren, Bo Li, Qi Wei, Zhiyong Zhu, He Zhou, Yunhao Tang, Zhihang Zhou, Linglong Peng, Yaxu Wang, Yahui Jiang

**Affiliations:** ^1^Department of Gastrointestinal Surgery, The Second Affiliated Hospital of Chongqing Medical University, Chongqing, China; ^2^Department of Clinical Nutrition, The Second Affiliated Hospital of Chongqing Medical University, Chongqing, China; ^3^Laboratory of Cancer Biology, Department of Oncology, University of Oxford, Oxford, United Kingdom; ^4^The Second Department of Gastrointestinal Surgery, Affiliated Hospital of North Sichuan Medical College, Nanchong, China; ^5^Department of Gastroenterology, The Second Affiliated Hospital of Chongqing Medical University, Chongqing, China

**Keywords:** sulfur microbial diet, colorectal cancer precursors, cancer prevention, epidemiology, dietary pattern

## Abstract

**Background:**

Sulfur microbial diet (SMD), related to the enrichment of sulfur-metabolizing gut bacteria, has been confirmed to be linked to an elevated risk of early-onset colorectal adenoma in young females. However, it remains unclear whether SMD is associated with the risk of colorectal adenoma in older people, who are at greater risk for colorectal cancer.

**Methods:**

All data on participants in this study were retrieved from the intervention arm of the Prostate, Lung, Colorectal, and Ovarian (PLCO) cancer screening test. Participants’ adherence to this dietary pattern was assessed using SMD score. Hazard ratios (HR) and 95% confidence intervals (CI) were adopted in Cox proportional hazards regression models to assess the link between SMD score and the incidence of colorectal adenoma in participants included in the study. Specific stratified analyses were constructed to assess whether this association changed in different conditions, whereas the robustness of the association was examined through sensitivity analyses.

**Results:**

The mean baseline age of participants was 62.1 (SD 5.2) years (range 54.0–75.0 years). During 19,468,589 person-years of follow-up, 992 colorectal adenoma cases were documented in a total of 17,627 included participants. In a fully adjusted model, an increased risk of colorectal adenoma was determined in participants in the highest quartile of SMD score in comparison with those in the lowest quartile (HR_quartile4_ vs. HR_quartile1_ = 1.23; 95% CI: 1.02, 1.47; *p* = 0.017 for trend). This positive association between SMD score and adenoma risk was more evident in participants who were current or former smokers (*p* = 0.029 for interaction).

**Conclusion:**

In this study, our results support a role for the SMD in the carcinogenicity of colorectal cancer precursors among older adults. Nevertheless, these results require validation through more research.

## Introduction

The second most prevalent cause of cancer mortality in the United States is colorectal cancer (CRC), which is considered to be the fourth most frequently diagnosed malignancy (1). Around 41,000 fatalities and 147,000 new cases of colorectal cancer are expected to be diagnosed by 2040 in the United States (2). Recently, research has increasingly indicated that the incidence of early-onset colorectal cancer is increasing rapidly, while the incidence of colorectal cancer after 50 years is gradually decreasing (3). However, it is undeniable that colorectal cancer diagnosed after the age of 50 years still constitutes the majority of newly diagnosed colorectal cancer. Approximately 90% of all colorectal cancer patients are diagnosed after 50 years of age (4). Identifying the high-risk factors for colorectal cancer in the elderly is still the focus of attention.

Colorectal traditional adenoma, as one of the recognized precursors of colorectal cancer, accounts for about 60–80% of sporadic CRC cases (5, 6). Although the majority of traditional adenomas can be removed by colonoscopy, recurrence is observed in nearly 50% of patients at 1 year of follow-up (7). Therefore, early identification of possible risk factors for traditional adenoma is of great significance to reduce the incidence of colorectal cancer. Recently, research has focused on the effects of diet, gut microbiota, and bacterial metabolites on the risk of colorectal cancer (8, 9). A sulfur microbial diet (SMD), which is related to the enrichment of sulfur-metabolizing gut microbiota, was constructed by Nguyen et al. through a large prospective study involving 51,529 U.S. males to investigate its effect on CRC risk (10). Specifically, SMD consists primarily of foods associated with CRC risk (such as decreased legumes and vegetables and an increase in processed meats), and long-term compliance to this diet was linked to a 43% greater risk of distal colon and rectum in this cohort (10). In a study involving a cohort of young female nurses aged 25–42, it was demonstrated that SMD is associated with a 58% increased risk of early-onset colorectal adenoma (11). Given the sex and occupational limitations of the populations included in the above studies, it remains unclear whether SMD is associated with the risk of colorectal adenoma in older individuals, who are at greater risk for colorectal cancer.

Hence, to determine the link between the SMD with the incidence of colorectal adenoma in the population older than 50, a prospective investigation in a large cohort of older adults was executed in the Prostate, Lung, Colorectal, and Ovarian (PLCO) Cancer Screening Trial.

## Materials and methods

### Study design

Details of the protocol and statistical analysis plan of the PLCO trial are available on this website,^1^ the recruitment plan for the study population and the objectives of the study had been thoroughly described in previous literatures (12–14). In brief, the PLCO trial recruited almost 155,000 men and women 55–74 years of age through competitively selected screening centers across the United States during the period of 1993–2001. In subsequent studies, the recruited population was assigned to the intervention or control arms in a 1:1 ratio by a reliable, secure randomized algorithm. The follow-up period continued until 2009–2018 to evaluate the effectiveness of early cancer screening (13). Each participant was required to submit a baseline questionnaire (BQ) covering self-administered risk factors. Additionally, the intervention arm participants were guided to fill in a dietary questionnaire (DQX) documenting daily dietary intake within 1 year and undergo screening programs including 60 cm flexible sigmoidoscopy (15). This trial was approved by the NCI Division of Cancer Prevention and Control, and written informed consent was obtained from the included participants.

### Definition of study cohort

**Figure 1:**
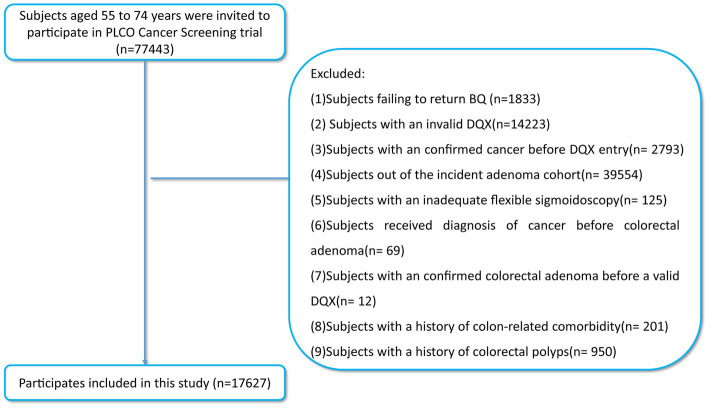
The flow chart of identifying subjects included in our study. PLCO, prostate, lung, colorectal, and ovarian; BQ, baseline questionnaire; DQX, dietary questionnaire.

The association between SMD and the incidence of colorectal adenoma was determined by executing a detailed nested case–control investigation limited to the intervention arm. Considering the purpose of this study, participants with the following conditions were excluded: (1) did not return complete baseline information; (2) did not return a valid DQX (including the completion date was either missing, or was later to the date of death; at least eight missing frequency responses were available; extreme calorie intake for each gender); (3) confirmed cancer before DQX entry; (4) out of the incident adenoma cohort (the identification: a negative screen at baseline and either a negative screen at T3/T5 or a positive screen at T3/T5 with a left-sided adenoma found on follow-up to the screen); (5) with an inadequate flexible sigmoidoscopy (insertion ≥50 cm with ≥90% of mucosa visualized); (6) received diagnosis of cancer before colorectal adenoma; (7) received a diagnosis of colorectal adenoma before returning a valid DQX; (8) had a history of colon-linked comorbidity (such as Gardner’s syndrome, ulcerative colitis, Crohn’s disease or familial polyposis); (9) had a history of colorectal polyps (Figure 1). Ultimately, the included participants of the study reached 17,627 in total (992 incident colorectal adenoma cases [655 males; 337 females] and 16,635 controls [9,213 males; 7,422 females]). This study was carried out with the permission of the United States NCI (CDAS project “PLCO-1070”).

### Data collection and SMD score calculation

This research involved data concerning demographics, lifestyle, and medical history, including age, sex, race, body mass index (BMI), smoking status, smoking pack-years, as well as the history of aspirin consumption, diabetes, hypertension, diverticulitis or diverticulosis, polyps and colonoscopy in the past 3 years retrieved through BQ. Dietary information, including total energy intake, and dietary food or nutrient intake, can be obtained through DQX. The DQX, which counted information on the intake frequency of 137 food items and consisted primarily of 61 Willett FFQ items, has been shown to provide effective, adequate information about the dietary intake of participants over a 1-year period (16–18). The supplemental questionnaire (SQX) was employed to investigate some items not reported in BQ, such as physical activity level, defined as the summarized weekly minutes of self-reported moderate to vigorous activity.

In an initial prospective study of 51,529 male from medical specialties, researchers developed the SMD score by assessing the correlation coefficient of sulfur-metabolizing bacteria abundance in the stools to food groups (10). The detailed components of SMD followed: (1) the positive correlation group included processed meats, liquor and low-calorie drinks; (2) the negative correlation group included beer, fruit juice, legumes, other vegetables and sweets. However, the specific components of SMD developed in another prospective study of 214,797 male and female from medical specialties by the same way were different, compared with the original study (11). In detail, the positive correlation group included low-calorie beverages, French fries, red meats, and processed meats, while negative correlation group included fruits, yellow vegetables, whole grains, legumes, leafy vegetables, and cruciferous vegetables. Considering the contribution of whole grains and sweets to colorectal cancer risk (19–21), we adjusted the specific components of SMD based on previous prospective studies (10, 11, 22). The adjusted SMD score was the sum of the quartile values from 1 to 4 of 8 components, consisting of processed meats, liquor and low-calorie drinks (higher quartiles of intake indicate higher scores); and beer, fruit drinks, legumes, whole grain, other vegetables (higher quartiles of intake indicate lower scores). Thus, the SMD score with a total score ranging from 8 to 32 could be used to assess adherence to this pattern of intake, with higher score indicating greater adherence. Specific food intake and corresponding distribution scores can be found in Supplementary Table S1. In subsequent studies, SMD score were categorized into quartiles.

### Assessment of conventional colorectal adenoma

As required in the PLCO trial, participants in the incident adenoma cohort are required to complete a screening colonoscopy at baseline. Subjects with negative results are allowed to enter a follow-up study and must complete at least one additional screening colonoscopy at T3 or T5. It means that none of the participants in this study had a diagnosis of colorectal adenoma at baseline, and all adenoma identifications during screening colonoscopy were confirmed by biopsied and further histological type. According to the current US guidelines for colonoscopy, conventional adenomas were categorized hierarchically: (1) any adenoma ≥1 cm, with high-grade dysplasia, or with tubulovillous or villous histology should be considered as advanced adenoma; (2) while only for the adenoma <1 cm and lacking advanced histology the diagnosis of non-advanced adenoma is considered (23).

### Statistical analysis

In this study, the data of some covariates were observed to be missing to varying degrees. Hence, for categorical and continuous variables with missing values reported at <5%, namely smoking status, pack-years, as well as the history of colonoscopy, aspirin usage, hypertension, diabetes, family history of colorectal cancer, and BMI, the missing data was imputed utilizing the modal value and the median, respectively (24). As for the variable “physical activity level” with 22.8% missing data, which were assumed to be randomly distributed, multiple imputations were done to complete them (25). The details of imputation values can be found in Supplementary Table S2.

**Figure 2:**
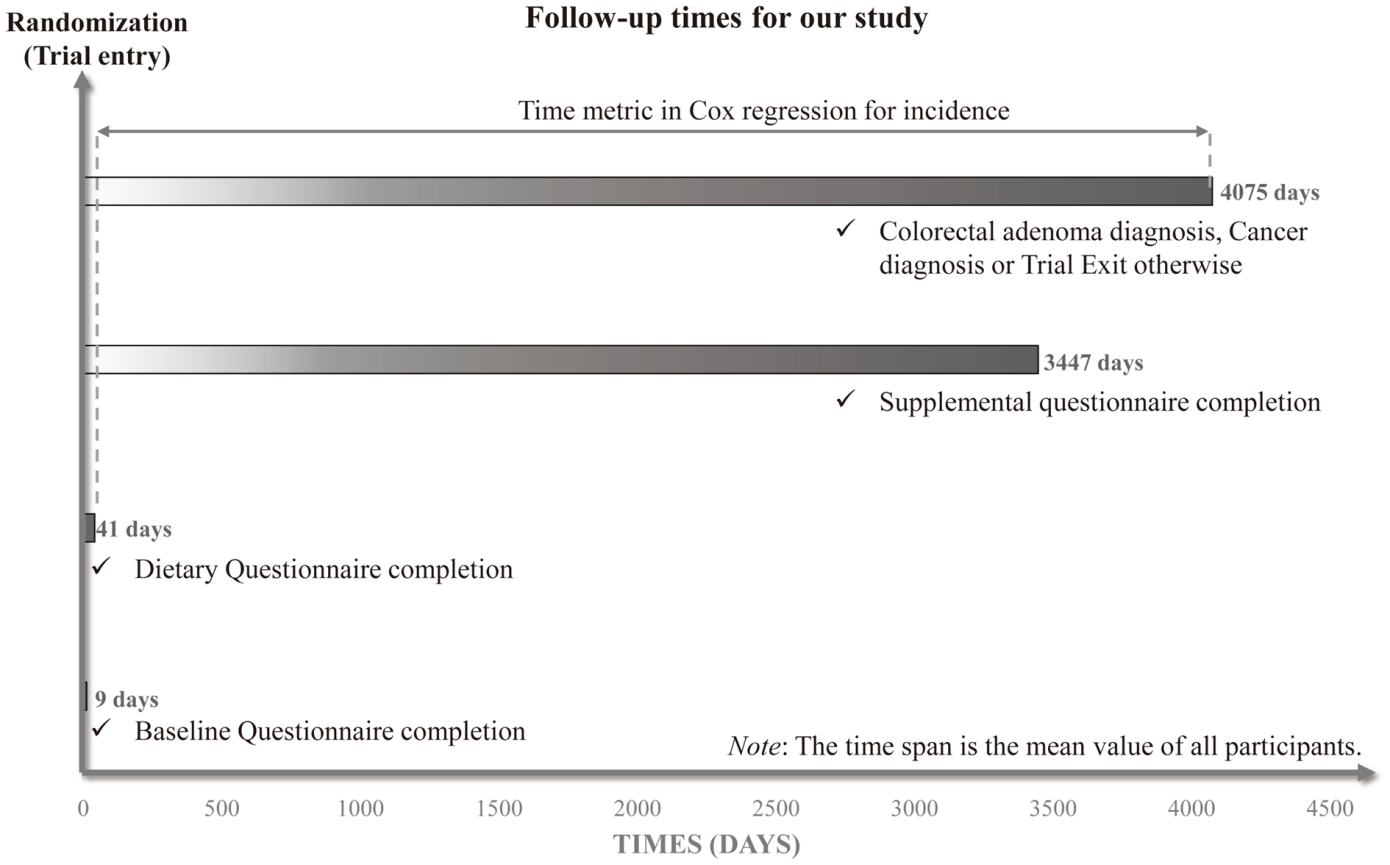
The timeline and follow-up scheme of our study.

The Cox proportional hazards regression model was constructed with follow-up time as the time variable for estimation of the 95% confidence interval (CIs) and hazard ratios (HRs) of the relationship between SMD score and the risk of colorectal adenoma. It should be emphasized that the main outcome event in this research was the confirmation of adenoma. In this research, the follow-up time was defined as the data from DQX completion to the diagnosis of adenoma, cancer, fatality, loss of follow-up, or end of follow-up (December 31, 2009), whichever came first (Figure 2).

To investigate the existence of a linear trend between quartiles of SMD score and the risk of colorectal adenoma, each participant in the quartile was assigned the median value of the quartile. This was then considered as a continuous variable in cox regression to order to get its *p*-value, with the reference group considered to be the lowest quartile. As per prior literature review and clinical judgment, sex, race, age, and education levels, total energy intake, BMI, aspirin usage, smoking status, smoking pack-years, as well as the history of hypertension, diverticulitis or diverticulosis, diabetes, colonoscopy, family history of colorectal cancer in past 3 years and physical activity level were adjusted as covariates in multiple regression analyses (11, 22, 26). Meanwhile, 12,916 participants with complete data were selected to test whether the analysis result was influenced by missing data imputation by repeating the same multiple regression analyses. To present colorectal adenoma risk across the full range of SMD score, a restricted cubic spline model with three knots at the 10th, 50th, and 90th was constructed in this study (27).

The influence of various factors on the observed association of SMD score with risk of colorectal adenoma was assessed by means of a series of pre-selected subgroup analyses including age (>65 vs. ≤65 years old), sex (male vs. female), BMI (≤30 vs. >30 kg/m^2^), smoking status (non-smokers vs. current/former smokers), smoking pack-years (≤median vs. >median years), family history of colorectal cancer (no vs. yes/possible), and history of aspirin consumption (no vs. yes). To verify the robustness of these findings, several sensitivity analyses were carried out: (1) exclusion of participants with a history of diverticulitis or diverticulosis; (2) exclusion of participants with a history of diabetes (more likely to have colorectal adenoma) (28); (3) exclusion of participants with a family history of colorectal cancer; (4) exclusion of colorectal adenoma cases observed within the first two and four years of follow-up to examine the likelihood of the observed association being caused by reverse causation; (5) Further adjusting model 2 for the Healthy Eating Index-2015 (HEI-2015) to determine if the observed link was diet quality-mediated.

All statistical analyses were completed through the software R 4.2.1. Furthermore, a two-tailed *p*-value less than 0.05 indicated the significance level.

## Results

### Population characteristic

A total of 17,627 participants [9,868 (55.08%) males and 7,759 (44.02%) females] were involved in the current analysis. The mean (standard deviation) baseline age of participants was 62.1 (5.2) years (range 54.0–75.0 years). The primary baseline features of participants per the quarters of the SMD score were depicted in tabular form (Table 1). In contrast with the lowest quartile (Q1), participants in the highest quartile (Q4) of SMD score tended to be younger, with more smoking pack-years, a higher BMI, a history of hypertension and diabetes, lower energy intake from diet, decreased physical activity level, and were less likely to be non-smokers, were regular users of aspirin, and had a family history of colorectal cancer. Additionally, in contrast to Q1, the participants of the Q4 of SMD score had increased intakes of processed meat, liquor, and low-calorie drinks but lower intakes of beer, fruit drinks, legumes, whole grains, and other vegetables.

**Table 1:** Baseline characteristics of study population according to overall sulfur microbial diet score.

		Quartiles of overall sulfur microbial diet score			
Characteristics	Overall	Quartile 1 (8–18)	Quartile 2 (19–20)	Quartile 3 (21–22)	Quartile 4 (23–32)
Number of participants	17,627	5,731	4,030	4,066	4,160
Sulfur microbial diet score	20.17 ± 3.18	16.45 ± 1.54	19.51 ± 0.50	21.47 ± 0.50	24.32 ± 1.40
Age	62.12 ± 5.17	62.50 ± 5.24	62.27 ± 5.16	62.03 ± 5.14	61.59 ± 5.06
Sex					
Male	9,868 (55.98%)	3,184 (59.28%)	2,279 (56.55%)	2,121 (52.16%)	2,284 (54.90%)
Female	7,759 (44.02%)	2,187 (40.72%)	1,751 (43.45%)	1,945 (47.84%)	1,876 (45.10%)
Race					
White	15,955 (90.51%)	4,758 (88.59%)	3,664 (90.92%)	3,710 (91.24%)	3,823 (91.90%)
Non-white	1,672 (9.49%)	613 (11.41%)	366 (9.08%)	356 (8.76%)	337 (8.10%)
Body mass index (kg/m^2^)	27.09 ± 4.55	26.66 ± 4.45	27.12 ± 4.50	27.22 ± 4.56	27.49 ± 4.67
Smoking status					
Never	9,379 (53.21%)	2,960 (55.11%)	2,184 (54.19%)	2,189 (53.84%)	2,046 (49.18%)
Current	970 (5.50%)	170 (3.17%)	199 (4.94%)	221 (5.44%)	380 (9.13%)
Former	7,278 (41.29%)	2,241 (41.72%)	1,647 (40.87%)	1,656 (40.73%)	1,734 (41.68%)
Smoking pack-years	13.93 ± 23.50	12.42 ± 21.47	13.60 ± 23.81	13.59 ± 23.44	16.54 ± 25.47
Drinking status					
No	3,704 (21.01%)	1,095 (20.39%)	859 (21.32%)	864 (21.25%)	886 (21.30%)
Yes	13,923 (78.99%)	4,276 (79.61%)	3,171 (78.68%)	3,202 (78.75%)	3,274 (78.70%)
Aspirin use					
No	9,418 (53.43%)	2,738 (50.98%)	2,166 (53.75%)	2,220 (54.60%)	2,294 (55.14%)
Yes	8,209 (46.57%)	2,633 (49.02%)	1,864 (46.25%)	1,846 (45.40%)	1,866 (44.86%)
Family history of colorectal cancer					
No	15,655 (88.81%)	4,818 (89.70%)	3,588 (89.03%)	3,610 (88.79%)	3,639 (87.48%)
Yes	1,521 (8.63%)	429 (7.99%)	348 (8.64%)	361 (8.88%)	383 (9.21%)
possibly	451 (2.56%)	124 (2.31%)	94 (2.33%)	95 (2.34%)	138 (3.32%)
History of diabetes					
No	16,563 (93.96%)	4,972 (92.57%)	3,780 (93.80%)	3,856 (94.84%)	3,955 (95.07%)
Yes	1,064 (6.04%)	399 (7.43%)	250 (6.20%)	210 (5.16%)	205 (4.93%)
History of hypertension					
No	12,236 (69.42%)	3,753 (69.88%)	2,807 (69.65%)	2,824 (69.45%)	2,852 (68.56%)
Yes	5,391 (30.58%)	1,618 (30.12%)	1,223 (30.35%)	1,242 (30.55%)	1,308 (31.44%)
History of colonoscopy or test for blood in stool					
No	9,963 (56.52%)	2,772 (51.61%)	2,241 (55.61%)	2,354 (57.89%)	2,596 (62.40%)
Yes	7,664 (43.48%)	2,599 (48.39%)	1789 (44.39%)	1712 (42.11%)	1,564 (37.60%)
Energy intake from diet (kcal/day)	2087.90 ± 798.59	2368.11 ± 811.86	2148.95 ± 784.18	1957.31 ± 744.88	1794.64 ± 711.34
Physical activity level (min/week)	129.78 ± 111.27	147.55 ± 116.04	133.63 ± 110.61	124.26 ± 108.82	108.50 ± 103.68
Healthy Eating Index-2015	66.54 ± 9.69	57.51 ± 7.93	65.19 ± 7.04	69.90 ± 6.49	75.46 ± 6.24
Components of SMD intakes					
Processed meat (g/day)	12.84 ± 16.49	9.71 ± 14.05	13.21 ± 17.53	13.48 ± 17.09	15.90 ± 17.10
Liquor (g/day)	15.35 ± 57.51	8.90 ± 42.78	13.99 ± 54.02	16.47 ± 50.98	23.89 ± 78.48
Low-calorie drinks (g/day)	86.89 ± 210.11	41.73 ± 127.35	73.99 ± 183.73	92.54 ± 213.41	152.18 ± 286.08
Beer (g/day)	117.05 ± 403.21	136.04 ± 450.09	123.59 ± 376.37	105.69 ± 405.64	97.31 ± 358.30
Fruit drinks (g/day)	17.62 ± 99.17	22.19 ± 99.42	20.00 ± 106.69	15.12 ± 99.87	11.87 ± 89.82
Legumes (cups/day)	0.10 ± 0.10	0.16 ± 0.14	0.10 ± 0.09	0.07 ± 0.06	0.05 ± 0.04
Whole grain (servings/day)	1.49 ± 1.04	2.10 ± 1.13	1.57 ± 0.96	1.23 ± 0.82	0.88 ± 0.65
Other vegetables (servings/day)	1.96 ± 1.08	2.69 ± 1.14	2.03 ± 0.96	1.67 ± 0.83	1.25 ± 0.62

Descriptive statistics are presented as (mean ± standard deviation) and number (percentage) for continuous and categorical.

### Association between SMD score and conventional colorectal adenoma risk

In this study, a total of 992 newly diagnosed conventional colorectal adenomas were documented during 19,468,589 person-years of follow-up, with an overall incidence rate of 0.51 cases per 1,000 person-years. The mean (standard deviation) follow-up length was 11.04 (3.50) years. In univariable analysis, in contrast with Q1, the participants of Q4 of SMD score were found to be at increased risk of colorectal conventional adenoma (HR_quartile4_: HR_quartile1_ = 1.28; 95% CI: 1.08, 1.51; *p* = 0.003 for trend Table 2). Subsequent to thorough adjustment for all possible confounders, the association of SMD score with the risk of conventional adenoma remained a positive one (HR_quartile4_: HR_quartile1_ = 1.23; 95% CI: 1.02, 1.47; *p* = 0.017 for trend Table 2). Notably, the repetition of the aforementioned analysis in a cohort of 12,916 participants with complete data resulted in similar data (HR_quartile4_: HR_quartile1_ = 1.24; 95% CI: 1.00, 1.54; *p* = 0.029 for trend; Supplementary Table S3).

**Table 2:** Hazard ratios of the association of SMD score with the risk of colorectal cancer precursors.

Quartiles of SMD core	Number of cases	Person-years	Incidence rate per 100 person-years (95% confidence interval)	Hazard ratio (95% confidence interval)		
				Unadjusted	Model 1^a^	Model 2^b^
Quartile 1 (8–18)	271	59980.23	0.452 (0.401, 0.509)	1.000 (reference)	1.000 (reference)	1.000 (reference)
Quartile 2 (19–20)	208	44620.56	0.466 (0.407, 0.534)	1.02 (0.85, 1.22)	1.01 (0.85, 1.22)	1.00 (0.83, 1.20)
Quartile 3 (21–22)	246	45063.23	0.546 (0.482, 0.618)	1.20 (1.00, 1.41)	1.21 (1.01, 1.43)	1.20 (1.00, 1.43)
Quartile 4 (23–32)	267	45021.86	0.593 (0.526, 0.668)	1.28 (1.08, 1.51)	1.27 (1.07, 1.50)	1.23 (1.02, 1.47)
P-trend				0.003	0.004	0.017

a Model 1: model 1 was controlled with age (continuous), sex (male, female), race (white, no-white) and education levels (college below, college graduate, postgraduate).

b Model 2: model 2 was additionally controlled with smoking status (never, current, former), pack-years of smoking (continuous), BMI (continuous), aspirin use (no, yes), history of hypertension (no, yes), history of diabetes (no, yes), family history of colorectal cancer (no, yes), total energy intake (continuous), history of diverticulitis or diverticulosis (no, yes), history of colonoscopy in past 3 years (no, yes), and physical activity level (continuous).

### Additional analyses

**Figure 3:**
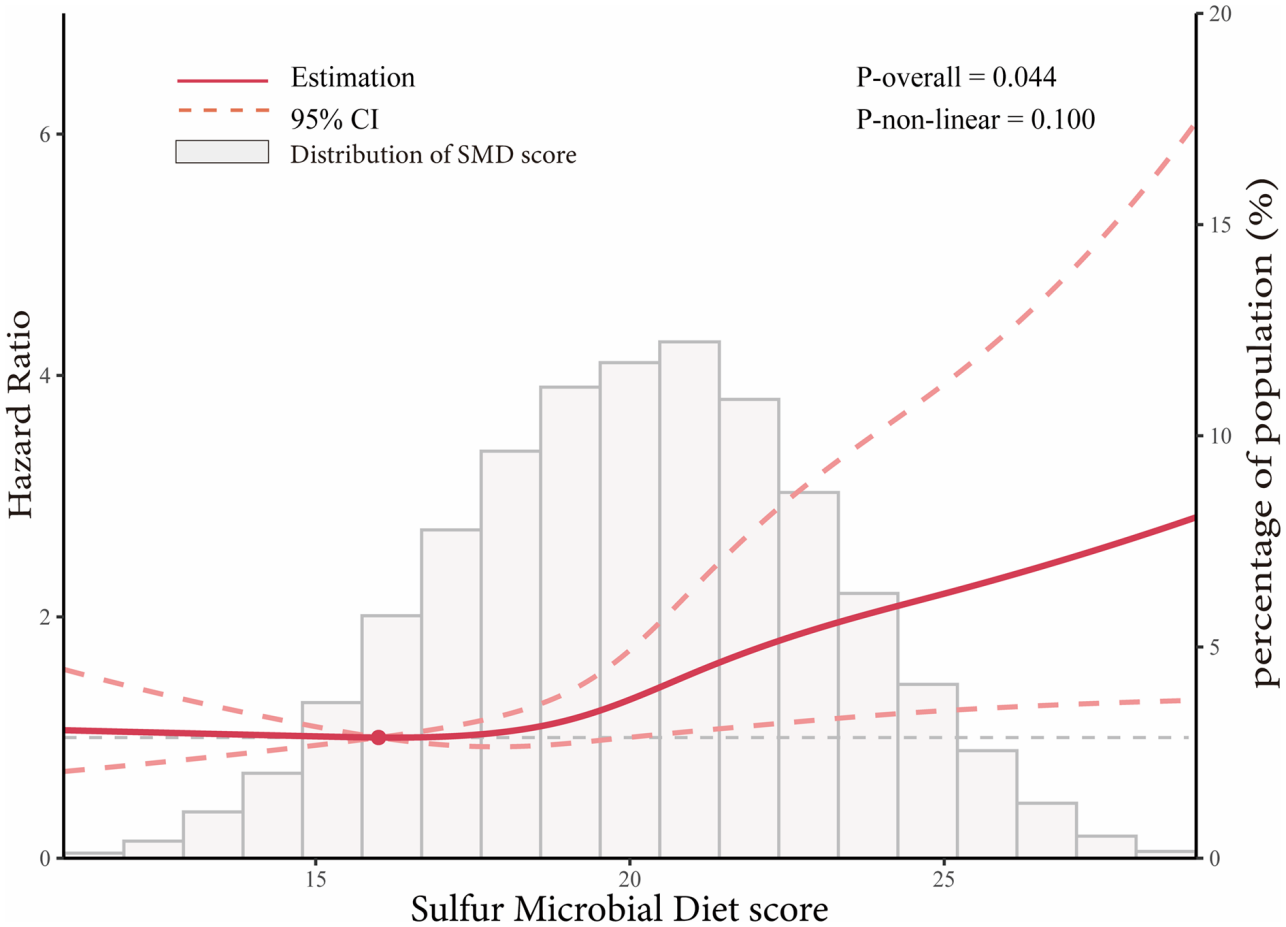
Dose–response analysis on the association of SMD score with the risk of colorectal adenoma (including total adenoma, advanced adenoma and non-advanced adenoma). Hazard ratios was adjusted for age, sex, race, education levels, smoking status, pack-years of smoking, BMI, aspirin use, history of hypertension, history of diabetes, family history of colorectal cancer, total energy intake, history of diverticulitis or diverticulosis, history of colonoscopy in past 3 years, and physical activity level.

In the whole study population, the linearity assumptions between SMD score and risk of colorectal conventional adenomas were validated by the restricted cubic spline (*p* = 0.100 for nonlinearity; Figure 3). The result of subgroup analysis in this study suggested that the status of smoking significantly modified the association between SMD score and incidence of conventional adenoma (*p* = 0.029 for interaction; Table 3). When compared with the lowest quartile of SMD score, HRs (95%CI) of incidence for the highest quartile of SMD score in the subsets of current or former smoker factors was 1.43 (1.12, 1.83). In addition, the positive association between SMD score and colorectal adenoma risk was depicted as more pronounced in males (HR_quartile4_: HR_quartile1_ = 1.28; 95% CI: 1.02, 1.62; Table 3) than in females (HR_quartile4_: HR_quartile1_ = 1.07; 95% CI: 0.77, 1.51; Table 3), though the interaction test was not statistically significant (concerning interaction *p* = 0.192). No other interactions were statistically significant (concerning interaction all *p* > 0.05; Table 3). The sensitivity analysis showed the initial associations of SMD score with risks of conventional adenoma were not impacted considerably through the exclusion of participants with specific preset conditions or further adjusting Healthy Eating Index-2015 (all *p* < 0.05 for trend; Table 4), which fully supports the stability of our findings.

**Table 3:** Subgroup analyses on the association of SMD score with the risk of colorectal cancer precursor.

Subgroup variable	Number of participates	Number of cases	HR _Quartile 4 vs. Quartile 1_ (95% CI)^a^	*P*_*-*interaction_
Age (years)				0.058
≤65	6,970	410	1.09 (0.86, 1.35)	
>65	2,561	128	1.65 (1.11, 2.45)	
Sex				0.192
Male	5,468	364	1.28 (1.02, 1.62)	
Female	4,063	174	1.07 (0.77, 1.51)	
Body mass index (kg/m^2^)				0.079
≤30	7,551	401	1.30 (1.04, 1.62)	
>30	1,980	137	1.01 (0.69, 1.47)	
Smoking status				**0.029**
Never	5,006	222	0.99 (0.74, 1.34)	
Current/former	4,525	316	1.43 (1.12, 1.83)	
Smoking pack-years				0.053
≤Medium	5,085	226	1.01 (0.75, 1.35)	
>Medium	4,446	312	1.40 (1.09, 1.80)	
Family history of colorectal cancer				0.989
No	8,457	482	1.18 (0.97, 1.45)	
Yes/possibly	1,074	56	1.37 (0.75, 2.51)	
History of aspirin consumption				0.071
No	5,032	280	1.02 (0.78, 1.33)	
Yes	4,499	258	1.47 (1.10, 1,91)	

^a^HRs were adjusted for age (years), sex (male, female), race (white, non-white), education levels (college below, college graduate, postgraduate), smoking status (never, current, former), pack-years of smoking (continuous), BMI (continuous), aspirin use (no, yes), history of hypertension (no, yes), history of diabetes (no, yes), family history of colorectal cancer (no, yes), total energy intake (continuous), history of diverticulitis or diverticulosis (no, yes), history of colonoscopy in past 3 years (no, yes), and physical activity level (continuous).

The bold values in Table 3 simply indicate statistical significance, with *p*-values less than 0.05.

**Table 4:** Sensitivity analyses on the association of SMD score with the risk of colorectal cancer precursors.

Categories	HR _Quartile 4 vs. Quartile 1_ (95% CI)^a^	*P*_-trend_
Primary analysis	1.23 (1.02, 1.47)	0.017
Excluded participants with history of diverticulitis or diverticulosis^b^	1.23 (1.02, 1.48)	0.016
Excluded participants with a history of diabetes^c^	1.23 (1.02, 1.49)	0.019
Excluded participants with family history of colorectal cancer^d^	1.22 (1.01, 1.48)	0.031
Excluded cases observed within the first 2 years of follow-up	1.23 (1.02, 1.47)	0.017
Excluded cases observed within the first 4 years of follow-up	1.27 (1.04, 1.56)	0.012
Further adjusted for Healthy Eating Index-2015^e^	1.23 (1.01, 1.50)	0.029

HR, hazard ratio; CI, confidence interval.

a HRs were adjusted for age (continuous), sex (male, female), race (white, no-white), education levels (college below, college graduate, postgraduate), smoking status (never, current, former), pack-years of smoking (continuous), BMI (continuous), aspirin use (no, yes), history of hypertension (no, yes), history of diabetes (no, yes), family history of colorectal cancer (no, yes), total energy intake (continuous), history of diverticulitis or diverticulosis (no, yes), history of colonoscopy in past 3 years (no, yes), and physical activity level (continuous).

b HR was not adjusted for history of diverticulitis or diverticulosis.

c HR was not adjusted for history of diabetes.

d HR was not adjusted for history of colorectal cancer.

e This covariate was treated as the continuous variable in multivariable Cox regression.

## Discussion

Based on a prospective large cohort study with adequate colonoscopy, the link between the SMD score and colorectal adenoma risk in the older population was assessed. According to the findings, following the SMD for a prolonged period of time was linked to an elevated risk of developing colorectal adenoma. The dose–response analysis also showed a linear trend of increasing the risk of colorectal adenoma with SMD score, suggesting that the risk of adenoma may increase in parallel with the increase in SMD score. The robustness of these findings was confirmed by subsequent sensitivity analysis. Our subgroup analysis showed that the positive association of SMD score with colorectal adenoma risk was only predominantly found in males, but not in females.

To develop a specific dietary pattern related to sulfur-metabolizing bacteria, Nguyen et al. analyzed the correlation of sulfur-metabolizing bacteria in stool samples with respective dietary components from 307 males (10). In his research, two main sulfur-metabolizing bacteria, *Erysipelotrichaceae bacterium 21_3* and *Bilophila wadsworthia* were identified to be associated with dietary (10), which has been previously confirmed to notably increase in the gut of patients with colorectal cancer or adenoma (29–31). Increasingly, research has shown that diet has a substantial influence on gut microbes, leading to an elevated risk of developing colorectal tumors via the colorectal adenoma-carcinoma sequence (32–34). It is well-recognized that sulfur-metabolizing microbes are involved in the conversion of dietary sulfur into hydrogen sulfide (H_2_S) in the gut, which contributes to the increased prevalence of colorectal tumors (35–38). Specifically, high concentrations of hydrogen sulfide in the intestine may raise the risk of colorectal tumors by damaging DNA in epithelial cells (39), promoting immune cell alterations associated with colorectal cancer (36), and damaging the bilayer of the intestinal mucosa (40, 41).

Notably, SMD score and smoking status depicted a significant interaction concerning the increased colorectal adenoma risk in the subgroup analysis (*p* = 0.029 for interaction). This means that participants who were former or current smokers would have an elevated risk of colorectal adenoma in comparison to participants who never smoked with the same SMD score, which was consistent with the previous study (22). One of the major risk factors for colorectal adenoma or colorectal cancer is considered to be smoking (42–44). Previous studies have shown that smoking leads to a significant shift in the gut microbiome of humans (45–47), which may be responsible for the increased risk of colorectal adenoma or colorectal cancer. However, microbial species changed by smoking mainly consisted of *Prevotella*, *Veillonella*, *Bacteroides*, and *Acidaminococcus* (46–48), which were different from those changed by the SMD. This may suggest that smoking may increase colorectal adenoma risk through different mechanisms, compared with a sulfur microbial diet. Recently, Bai et al. explored the mechanism of smoking and gut microbial-mediated colorectal tumorigenesis in mice, the result demonstrating that smoking can promote colonic tumorigenesis by modulating the components of the gut microbiota and inducing dysbiosis of the gut microbiota (49). Smoking may promote colorectal tumorigenesis which results in impairing the gut barrier function, promoting inflammation in colon tumorigenesis, and enhancing oncogenic *MAPK/ERK* signaling in colonic epithelium (49), which is partially similar to the mechanism of H_2_S promoting colorectal tumors (36, 40, 41). Smoking may modulate the abundance of microbiota other than sulfur-metabolizing microorganisms in the gut to have some synergistic effect on the increased colorectal adenoma risk induced by the SMD, which may provide a possible explanation for these results. However, this explanation needs to be confirmed by further investigating the interactions between the different microbiota mentioned above.

Intriguingly, our subgroup analysis revealed a more pronounced positive association between adherence to SMD and the risk of colorectal adenomas in males. Several potential explanations can shed light on this observation. Firstly, in our study cohort, males constituted a higher proportion of smokers, comprising approximately 66% of all current or former smokers. Moreover, our subgroup analysis indicated a significant interaction between smoking and SMD adherence in increasing the risk of colorectal adenomas. Given that smoking is a known contributor to colorectal adenoma risk (50), this difference in smoking prevalence between genders may contribute to the sex-specific association observed in colorectal adenoma incidence. On another note, Liu et al. demonstrated that adherence to SMD was linked to an increase risk of obesity (51). Their gender-specific stratified analysis further suggested a more substantial positive association between SMD adherence and obesity risk in males compared to females (51). Considering that obesity is a significant risk factor for colorectal cancer and adenoma (52, 53), the variation in the association between obesity and adherence to the SMD pattern across genders may be the reason why the association between SMD and colorectal adenoma risk is more significant in males.

This study has several strengths. First, unlike previous studies that conducted their study only on health professionals (10, 11, 22), the population in this study was more representative because an almost equal proportion of male and female participants were involved, with no occupational restrictions, who received the same care in different practice settings across the United States. Second, participants with inadequate flexible sigmoidoscopy were excluded, which guaranteed the effectiveness of the colonoscopy. Third, considering the inherent influence of colon-related complications with a genetic predisposition on the incidence of colorectal cancer (such as Crohn’s disease, Gardner’s syndrome, ulcerative colitis, or familial polyposis) (54–57), participants with colon-related complications were excluded to minimize the interference of genetic factors on the study results. Notably, this research confirms for the first time that SMD is linked to an elevated risk of colorectal adenoma in the older individuals. Given the higher risk of colorectal tumors in the elderly population compared to the younger population (4), this research will provide a new dietary guideline for them to minimize the incidence of CRC in this high-risk population.

This research is restricted in some aspects. The microbiota in the stool samples of participants was not analyzed due to some limitations, therefore, the shift in the intestinal microbiota of participants could not be guaranteed to be consistent with previous studies. However, SMD-related analyses have been adequately validated in several various study cohorts (10, 11, 22), making this deficiency acceptable. In addition, the dietary intake of SMD using DQX was calculated only once at baseline, rather than calculating the cumulative mean at long-term follow-up, which may lead to nondifferential bias. However, based on a classical assumption in nutrition, the exposure measured at baseline is more reflective of the daily dietary habits of participants in the years before and following inclusion in the study (24). Hence, these calculations for the dietary intake of participants can be considered valid.

## Conclusion

To summarize, our study findings revealed a positive correlation between SMD score and conventional colorectal adenomas risk in an elderly population in the United States, with a median follow-up of 11 years. Furthermore, this positive association is more significant in males. Smoking may have a synergistic effect on the positive association between SMD and colorectal adenoma by modulating intestinal microbiota, which differed from the sulfur-metabolizing bacteria, and the exact mechanism needs to be elucidated by subsequent in-depth studies on the mechanism of intestinal microbiota interactions.

## Data availability statement

The raw data supporting the conclusions of this article will be made available by the authors, without undue reservation.

## Ethics statement

The studies involving human participants were reviewed and approved by the Institutional Review Board of the National Cancer Institute. The patients/participants provided their written informed consent to participate in this study.

## Author contributions

HH, YW, YJ, and YX contributed to the study design and data analysis. YX and YJ contributed to the data interpretation and writing of the manuscript. YX, LX, ZX, XR, BL, YJ, ZyZ, HZ, YT, HL, QW, ZhZ, HH, and HG contributed to the data collection, and data curation of the present analysis. LP, LX, YW, HH, and HG assisted with statistical analysis and funding acquisition. YJ and HH made significant contributions to the revised manuscript. All of the authors reviewed or revised the manuscript. All authors contributed to the article and approved the submitted version. The work reported in the paper has been performed by the authors, unless clearly specified in the text.

## Funding

This work was supported by the General Project of Chongqing Natural Science Foundation, Chongqing Science and Technology Commission, China [cstc2021jcyj-msxmX0153 (LP)], [cstc2021jcyj-msxmX0112 (YW)], and [CSTB2022NSCQ-MSX1005 (HG)], and Kuanren Talents Project of the Second Affiliated Hospital of Chongqing Medical University in China [kryc-yq-2110 (HG)]. The funders had no role in the study design or implementation; data collection, management, analysis or interpretation; manuscript preparation, review or approval; or the decision to submit the manuscript for publication.

## Conflict of interest

The authors declare that the research was conducted in the absence of any commercial or financial relationships that could be construed as a potential conflict of interest.

## Publisher’s note

All claims expressed in this article are solely those of the authors and do not necessarily represent those of their affiliated organizations, or those of the publisher, the editors and the reviewers. Any product that may be evaluated in this article, or claim that may be made by its manufacturer, is not guaranteed or endorsed by the publisher.

## Footnotes

^1^https://cdas.cancer.gov/plco/
