# Supplementary material for: Association between sulfur microbial diet and the risk of colorectal cancer precursors in older adults
Source: Front Nutr. 2023 Aug 14;10:1167372. doi: 10.3389/fnut.2023.1167372 (PMC10461007; doi:10.3389/fnut.2023.1167372)
Supplement: Supplementary file 1 [file Data_Sheet_1.pdf]

Supplementary Material

Association Between Sulfur Microbial Diet and the Risk of Colorectal Cancer Precursors in Older Adults

Yi Xiao[1, †], Hongmei He[1, †], Ling Xiang[2], Haitao Gu[1], Zhiquan Xu[1], Haoyun Luo[1], Xiaorui Ren[1], Bo Li[1], Qi Wei[1], Zhiyong Zhu[1], He Zhou[3, 4], Yunhao Tang[1], Zhihang Zhou[5], Linglong Peng[1], Yaxu Wang[1, *] and Yahui Jiang[1, *]

[1] Department of Gastrointestinal Surgery, The Second Affiliated Hospital of Chongqing Medical University, Chongqing, China.

[2] Department of Clinical Nutrition, The Second Affiliated Hospital of Chongqing Medical University, Chongqing, China.

[3] Laboratory of Cancer Biology Department of Oncology, University of Oxford, Old Road Campus Research Building, Oxford, UK.

[4] The Second Department of Gastrointestinal Surgery, Affiliated Hospital of North Sichuan Medical College, Nanchong, Sichuan, China.

[5] Department of Gastroenterology, The Second Affiliated Hospital of Chongqing Medical University, Chongqing, China.

*** Correspondence authors: Yaxu Wang and Yahui Jiang (Department of Gastrointestinal Surgery), The Second Affiliated Hospital of Chongqing Medical University, No.288 Tianwen Avenue, Nan'an District, Chongqing, 400010, China. fax: +86 023 6288 7521. E-mail: 300897@hospital.cqmu.edu.cn (Yaxu Wang); 304792@hospital.cqmu.edu.cn (Yahui Jiang)**

[†] These authors contributed equally: Yi Xiao, Hongmei He.

Supplementary Tables

Supplementary Table 1. Criteria for determining dietary Sulfur Microbial Diet score

	Energy-adjusted dietary intakes of individual components							
Points	Processed meat (g/day)	Liquor (g/day)	Low-calorie drinks (g/day)	Beer (g/day)	Fruit drinks (g/day)	Legumes (cups/day)	Whole grain (servings/day)	Other vegetables (servings/day)
4	≥15.91	≥3.90	≥49.22	≤0	≤0	≤0.04	≤0.75	≤1.20
3	7,22–15.90	0.52–3.89	5.67–49.21	0.01–4.01	0.01–1.81	0.05–0.07	0.76–1.28	1.21–1.76
2	3.13–7,21	0.01–0.51	1.65–5.66	4.02–31.80	1.82–3.68	0.08–0.12	1.29–1.97	1.77–2.50
1	≤3,12	0	≤1.64	≥31.81	≥3.69	≥0.13	≥1.98	≥2.51

Supplementary Table 2. Distribution of covariates with missing data before and after imputation *

Variable	Before imputation	After imputation	Number (%) with missing data
Family history of colorectal cancer			113 (0.64%)
No	15655 (88.81%)	15542 (88.74%)	
Yes	1521 (8.63%)	1521 (8.68%)	
Possibly	451 (2.56%)	451 (2.58%)	
Body mass index (kg/m^2)	27.09±4.55	27.22±4.79	164 (0.93%)
Smoking status			4 (0.02%)
Never	9379 (53.21%)	9375 (53.20%)	
Current	970 (5.50%)	970 (5.50%)	
Former	7278 (41.29%)	7278 (41.30%)	
Smoking pack-years	13.93±23.50	14.06±23.57	156 (0.89%)
Aspirin use			54 (0.31%)
No	9418 (53.43%)	9364 (53.29%)	
Yes	8209 (46.57%)	8209 (46.71%)	
History of hypertension			8 (0.05%)
No	12236 (69.42%)	12228 (69.40%)	
Yes	5391 (30.58%)	5391 (30.60%)	
History of diabetes			3 (0.02%)
No	16563 (93.96%)	16560 (93.96%)	
Yes	1064 (6.04%)	1064 (6.04%)	
History of Colonoscopy or Test for Blood in Stool			16 (0.09%)
No	9963 (56.52%)	16786 (95.32%)	
Yes	7664 (43.48%)	825 (4.68%)	
Physical activity level (min/week)	129.78±111.27	131.18±124.30	3927 (22.28%)

Supplementary Table 3. Hazard ratios of the association of SMDs with the risk of colorectal adenoma in 12916 participants with complete data

Quartiles of SMDs	Number of cases	Person-years	Incidence rate per 100 person-years (95% confidence interval)	Hazard ratio (95% confidence interval)		
				Unadjusted	Model 1 [a]	Model 2 [b]
Quartile 1 (8-18)	194	45200.61	0.429 (0.373, 0.494)	1.000 (reference)	1.000 (reference)	1.000 (reference)
Quartile 2 (19-20)	149	33453.29	0.445 (0.380, 0.523)	1.02 (0.83, 1.27)	1.02 (0.83, 1.27)	1.01 (0.81, 1.25)
Quartile 3 (21-22)	181	33501.22	0.540 (0.467, 0.625)	1.23 (1.01, 1.51)	1.26 (1.03, 1.54)	1.25 (1.01, 1.53)
Quartile 4 (23-32)	190	33397.78	0.569 (0.494, 0.655)	1.28 (1.05, 1.57)	1.28 (1.04, 1.56)	1.24 (1.00, 1.54)
$P_{\text{-trend}}$				0.008	0.010	0.029

SMD, sulfur microbial diet.

[a] Model 1: model 1 was controlled with age (continuous), sex (male, female), race (white, no-white) and education levels (college below, college graduate, postgraduate).

[b] Model2: model 2 was additionally controlled with smoking status (never, current, former), pack-years of smoking (continuous), BMI (continuous), aspirin use (no, yes), history of hypertension (no, yes), history of diabetes (no, yes), family history of colorectal cancer (no, yes), total energy intake (continuous), history of diverticulitis or diverticulosis (no, yes), history of colonoscopy in past 3 years (no, yes), and physical activity level (continuous).
